# Effect of Ultrasound and Salt on Structural and Physical Properties of Sodium Alginate/Soy Protein Isolates Composite Fiber

**DOI:** 10.3390/foods12234275

**Published:** 2023-11-27

**Authors:** Xinyue Zeng, Bing Cui, Bin Zhou, Hongshan Liang, Di Wu, Jing Li, Bin Li

**Affiliations:** 1 College of Food Science and Technology, Huazhong Agricultural University, Wuhan 430070, China; zxywyyx1@163.com (X.Z.); cbtz0817@webmail.hzau.edu.cn (B.C.); lianghongshan@mail.hzau.edu.cn (H.L.); lijingfood@mail.hzau.edu.cn (J.L.); 2 Key Laboratory of Environment Correlative Dietology, Ministry of Education, Huazhong Agricultural University, Wuhan 430074, China; 3 Key Laboratory of Fermentation Engineering, Ministry of Education, National “111” Center for Cellular Regulation and Molecular Pharmaceutics, Wuhan 430068, China; zhoubin4111@163.com; 4 Hubei Key Laboratory of Industrial Microbiology, School of Biological Engineering and Food, Hubei University of Technology, Wuhan 430068, China

**Keywords:** ultrasound, meat analog fiber, wet-spinning, physical characterization

## Abstract

Recently, there has been a growing interest in advancing plant-based or cultured meat substitutes as environmentally and ethically superior alternatives to traditional animal-derived meat. In pursuit of simulating the authentic meat structure, a composite fiber composed primarily of soy protein isolates (SPIs) was fashioned, employing a fiber-based plant-based analog meat construct. To refine the spinning process and enhance fiber quality, we employed ultrasound treatment, a physical modification technique, to scrutinize its influence on SPI protein structure. This inquiry extended to the examination of the interplay between sodium alginate (SA) and SPI, as well as the impact of salt ions on the SA and ultrasound soy protein isolates (USPI) interaction. A comprehensive exploration encompassing ultrasound treatments and salt concentrations within the composite solution, along with their repercussions on composite fiber characterization, with a rise in negative zeta potential value, states the ultrasound treatment fosters protein aggregation. Moreover, the introduction of salt augments protein aggregation as salt content escalates, ultimately resulting in a reduced structural viscosity index and improved spinnability. The presence of Ca^2+^ ions during the coagulation process leads to interactions with SA. The involvement of ultrasound prompts the exposure of hydrophilic amino acid segments in the protein to water, leading to the development of a more porous structure. Solely under the influence of ultrasound, the fiber exhibits 5% higher water-holding capacity and superior mechanical properties while maintaining comparable thermal stability.

## 1. Introduction

In recent years, there has been a burgeoning interest in the development of plant-based or cultured meat alternatives as a more sustainable and ethical option compared to traditional animal-based meat [1]. Despite the growth in popularity of meat alternative products made from legumes, such as patties, nuggets, and kebabs [2,3], there were still limitations remaining in terms of nutrition, texture, and mouthfeel [4]. These limitations arise from the fibrousness of animal meat, which results from the collagen fibrils (perimysium and endomysium) entrapping bundles of muscle fiber [3]. To address this challenge, various processing techniques can be employed, including wet or electro-spinning, extrusion, 3D printing, and high-temperature conical shear cells [2]. The wet-spinning method, as a bottom-up method, is one of the most common manufacturing techniques for the fabrication of fibrous structures, and usually provides the feasibility to produce individual fiber that can be further bundled and stretched to orient fiber microstructure [2]. Through 4 main stages: dissolution, extrusion, coagulation, and collection, the wet-spinning method will be able to produce fiber-based biomaterial that is founded on the concept of solvent exchange between the polymer solution and the coagulation bath [5]. To adapt the wet-spinning method for use in the food industry, to accommodate edible materials into the production and with a similar manufacturing process, it would be ideal to create large-scale, low-cost, high-yield plant-based analog meat microfibers [5,6,7].

Two key factors play a vital role in the wet-spinning process: the polymer solution and the coagulation bath. The polymer concentration, molecular weight, additives in the polymer solution, and the processing temperature are important parameters that would affect the spinning process and fiber quality [5]. The polymer in the solution is extruded out of the nozzle and in contact with the coagulation bath, it begins to coagulate and solidify which is induced by solvent/non-solvent exchange [5,6]. The coagulation process is a diffusion-controlled phase separation process that can produce fiber. The porous structure on the surface of the fiber is usually caused by coagulation, where higher processing temperature results in increased mutual diffusion coefficients and, therefore, more porous structure [5].

Proteins and polysaccharides can be used as ingredients for spinning. By successfully combining sodium alginate (SA) and soy protein isolates (SPI), we are able to produce a high protein-content plant-based analog meat fiber [8]. Limited by the high protein content and large molecule size, the fluidity and spinnability of the fiber need to be improved. Considering the food safety aspect of the product and minimizing modifications to the raw material, modulation of the electrostatic interaction between protein molecules and reduced protein molecule size physiochemically were applied. Recent studies on the physical modification of plant-based protein mainly stated five approaches: conventional heat treatment, high pressure, sonication, extrusion, and cold atmospheric plasma processing [9]. Sonication is a green, sustainable, novel technique that has several advantages compared to other modification methods. In particular, ultrasound (US) emits an acoustic sound wave above 20 kHz, which the wave can be affected by pressure and displacement. The radicals and superoxide produced during the protein US modification process may induce structural changes caused by the disruption of non-covalent bonds [9,10]. Due to the above, sonification can apply a total effect on the protein structure that would decrease protein viscosity and reduce intrinsic viscosity [11]. Studies have shown that US treatments had positive impacts on the structural, physicochemical, and functional properties of plant protein, including solubility, emulsification, gelation, water-holding capacity, foaming, and oil absorption capacity [12,13,14,15,16,17]. 

In this current work, we aim to optimize the spinning process and improve the performance and mechanical strength of the fiber through the effect of US treatment on the SPI, and the addition of ions to influence the ionic environment of the protein. Scanning electron microscope, Fourier-transform infrared spectroscopy, and differential scanning calorimetry were used to characterize the morphology, secondary structure, and thermal properties of the modified composite fiber, respectively. Water-holding capacity, mechanical properties, and moisture distribution were performed to investigate the properties of the sodium alginate and US pre-treated soy protein isolate (USPI) composite fibers. In this study, our work will redesign the spinning medium based on US modification on the plant protein, and further improve the quality and water retainability of obtained fiber. 

## 2. Materials and Methods

### 2.1. Materials 

Defatted soybean flour was purchased from Shandong Yuwang Industrial Co., Ltd. (Yucheng, Shandong Province, China). Food-grade sodium alginate (Mn = 357,475 Da, Mn/Mw = 1.392, M/G =0.32) was purchased from Qingdao Haizhilin Biotechnology Development Co., Ltd. (Qingdao, China). Analytical pure hydrochloric acid (HCl), sodium hydroxide (NaOH), calcium chloride (CaCl_2_), sodium chloride (NaCl), and potassium chloride (KCl) were all purchased from Sinopharm Chemical Reagent Co., Ltd. (Wuhan, China).

### 2.2. USPI Preparation

The SPI was extracted with methods adopted from [18,19]. The protein content of SPI determined by the Kjeldahl method was 90.36 ± 0.35% (N × 6.25). The SPI powder was dispersed in distilled water with a total solids content of 10% (*w/v*), slowly stirred for 2 h at room temperature, and stored in the fridge overnight at 4 °C. The 100 mL SPI dispersion was treated using a 20 kHz angle sensor sonicator (FB705, Thermo Fisher Scientific Inc., Waltham, MA, USA) with a 12 mm titanium probe. Place it in a 100 mL flat-bottom cylindrical flask, and soak it in an ice water bath. Samples were treated at 80% amplitude for 5 min (pulse time 5 s, intermittent 5 s), after sonication all samples were lyophilized and stored at room temperature in a sealed container.

### 2.3. Preparation of SA/USPI Composite Solutions

At ambient temperature, a specified quantity of SA was completely dispersed and dissolved in distilled water, resulting in an SA solution with a mass fraction of 7 wt%. Simultaneously, the sonicated SPI was dissolved in distilled water and stirred for 2 h to produce a USPI solution with a mass fraction of 16 wt%. All solutions were stored at 4 °C overnight to ensure complete hydration. The pH values of the SA and SPI solutions were adjusted to 7, respectively. Next, equal amounts of SA solution and SPI solution (labeled with rhodamine B) were combined. 

Varying amounts of NaCl or KCl were added to create composite spinning solutions with different salt contents. Control samples without added salt were also prepared. The solutions were mechanically stirred and degassed by centrifugation (10,000× *g*) for 10 min.

### 2.4. Confocal Laser Scanning Microscopy (CLSM)

The solution morphology was observed using a confocal laser scanning microscope (CLSM, FV3000, Olympus, Shinjuku-ku, Tokyo, Japan). Modifications on the method were made based on previous study [20]. Nile Blue A was used to label SPI. The stained USPI solution was then mixed with SA solution and thoroughly stirred for 1 min. The stained samples were placed on a glass slide with a coverslip, and all images were obtained at a wavelength of 488 nm using CLSM at a magnification of 100×.

### 2.5. Zeta Potential

The zeta potential was measured using Zetasizer Nano-ZS90 (Grovewood Rd., Malvern, WR14 1XZ, U.K.) based on dynamic light scattering (DLS) technology. Each sample was diluted 1000 times with the corresponding solution and tested at 25 °C, in triplicate. The final result was recorded by its average.

### 2.6. Rheological Measurement

The rheological properties of the SA/USPI composite solution were measured by the DHR-2 rheometer (DHR-2, TA Instruments, New Castle, DE, USA) with a parallel plate geometry (40 mm diameter, 1000 μm gap) equipped. The steady-state flow behavior of the sample was measured at a shear rate of 0.1 s^−1^–100 s^−1^ at 25 °C. Sample flow behavior was characterized by the power-law equation with the following formula [21]:

(1) $$\tau =K\cdot \gamma^{n}$$

(2) $$n=\frac{\mathrm{dlg}\;\tau }{\mathrm{dlg}\;\gamma }$$

where K is the apparent viscosity constant, τ is the shear stress, γ is the shear rate, and n is the non-Newtonian index.

The spinning performance of the composite solution is determined by the following formula:

(3) $$\Delta \eta =-\left[\frac{d(\mathrm{lg}\eta )}{d(\sqrt{\gamma })}\right]{\times 10}^{2}$$

where △η is the structural viscosity index.

### 2.7. Preparation of SA/USPI Composite Fibers

**Figure 1:**
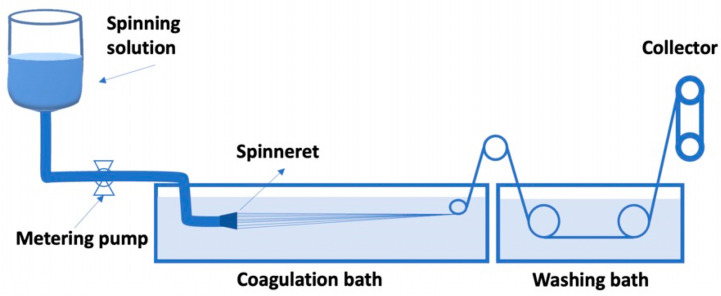
The process flow diagram of SA/USPI composite fibers produced by wet spinning.

The process flow of the wet-spinning method-prepared SA/USPI composite fiber is shown in Figure 1. The SA/USPI composite spinning solution was extruded from 75-hole spinnerets (0.12 mm diameter) into a 3% (*w*/*w*) CaCl_2_ solidification bath at 25 °C with a metering pump (speed of 6 mL/min) to obtain SA/USPI composite fiber bundles. The fiber bundles were subsequently washed in a washing tank and collected by a collecting roller. The naming of the SA/USPI composite spinning fiber corresponded to its matching composite solution. 

### 2.8. Scanning Electron Microscope (SEM)

The SA/USPI composite fibers were flash-frozen in liquid nitrogen immediately after preparation to obtain the cross-section. The frozen fibers were then transferred to a vacuum freeze dryer. The surface and cross-sectional morphology of the fiber were analyzed using a scanning electron microscope (SEM) (JSM-6390LV, Jeol, Tokyo, Japan) with an accelerating voltage of 5 kV after sputter-coating with Au. All images were recorded under a magnification of 500×.

### 2.9. Fourier-Transform Infrared Spectroscopy (FT-IR)

The fiber powder obtained from the wet-spinning process was blended with KBr with a mass ratio of 1:50, then grounded and pressed into pellets for Fourier transform infrared spectrometer (FTIR) (Nicolet iS50, Thermo Fisher Scientific Co., Waltham, MA, USA). The infrared spectra of the corresponding samples were obtained by scanning 32 times at a resolution of 4 cm^−1^ in the wavenumber range of 500–4000 cm^−1^. 

The spectra were processed via OMNIC 8.0 (Thermo Fisher Scientific Co., Waltham, MA, USA), and Peak Fit v4 software (SeaSolve Software Inc. San Jose, CA, USA) were used to determine the shift and peak area ratio of absorption peaks [22]. Baseline correction and Fourier self-deconvolution were performed. Second-order derivation and Gaussian curve fitting were conducted. According to the area of each peak in the range of 3700–3000 cm^−1^ and the peak area in the amide I region (1600–1700 cm^−1^), the content of different types of hydrogen bonds and the protein secondary structure content were calculated, respectively.

### 2.10. Differential Scanning Calorimetry (DSC)

The thermal properties of lyophilized fibers were determined by differential scanning calorimetry (DSC) (DSC200F3, New Castle, TA Instruments, USA). DSC analysis of all samples was performed in an N_2_ flow at 50–350 °C at a heating rate of 10 °C/min, and all tests were performed under N_2_ flow with purge gas flow at 20 mL/min and protecting gas flow at 60 mL/min [23].

### 2.11. Water Holding Capacity (WHC)

The sample (2 g) was wrapped in filter paper and placed in a centrifuge tube and centrifuged at 2000 g for 1, 3, 5, 10, 15, and 20 min. Excess moisture was removed from the surface of fresh fibers with filter paper before centrifugation. The formula for calculating WHC is as follows [8]:

(4) $$\mathrm{WHC}\;(\%)=\frac{W_{a}}{W_{b}}\times 100$$

In the formula:

W_a_ is the total weight (g) of the fresh fiber after centrifugation,

W_b_ is the total weight (g) of the fresh fiber before centrifugation.

### 2.12. Low-Field Nuclear Magnetic Resonance (LF-NMR)

The moisture distribution of fresh fibers was measured using a low-field nuclear magnetic resonance (LF-NMR) analyzer (MesoQMR23060H, Niumag Electric Corporation, Shanghai, China) with a modified method adapted from Jiang et al., 2022. Before testing, excess water was removed from the fiber bundle surface using filter paper. For testing, 2 g of the sample was wrapped in polytetrafluoroethylene film and placed in a glass tube with a diameter of 15 mm, which was then inserted into the NMR probe. The resonant frequency used was 21 MHz and the operating temperature was 32 °C. The transverse relaxation time (T_2_) was determined using the Car-Purcell-Meiboom-Gill (CPMG) sequence with the following parameters: SW = 100 kHz; RG = 20 dB; Nech = 3000; TE = 0.2 ms; NS = 4; TW = 2000 ms. The T_2_ software was used to adapt CPMG to T_21_, T_22_, and T_23_, and calculate the peak ratio. And P_21_, P_22_, and P_23_ is corresponding to the proportion of water distribution.

### 2.13. Mechanical Property Analysis

According to the previous methods [24,25,26] with modification, the strength of analog fibers was tested using a permeable myofiber testing system (a set of equipment specifically designed to measure the tension of muscle fibers in animals). A fresh fiber is fixed using cellulose acetate glue between the micromanipulators, and the fiber length is rapidly changed by force sensors and a computer-controlled feedback system (Aurora Scientific 802 B, Aurora, ON, Canada). In a sample cell filled with distilled water measure 3 fibers from each sample, then stretch each fiber 6 times, recording the force for every L/Ls step (i.e., stretch length/relaxation length). The obtained results were analyzed statistically.

### 2.14. Statistical Analysis

The data collation and statistical analysis were carried out using IBM SPSS Statistics 22.0 software (IBM Corp., New York, NY, USA). Origin 2022b software (OriginLab Corporation, Northampton, MA, USA) was utilized for mapping purposes. The significance of the results was assessed through one-way ANOVA and 95% confidence intervals. All samples were tested in triplicate.

## 3. Results and Discussions

The primary objective of this current investigation is to explicate the consequences of ultrasound treatment on the composite solution and its impact on the spinning process. Additionally, we seek to enhance our comprehension of the interrelationship between polysaccharides and proteins, as well as the ramifications of salt ions on the fiber formation process and the ultimate quality of the resulting fibers.

### 3.1. Effect of Ultrasound Treatments and Salt on the SA/USPI Composite Solution 

#### 3.1.1. Micro-Morphology and Zeta Potential Analysis

**Figure 2:**
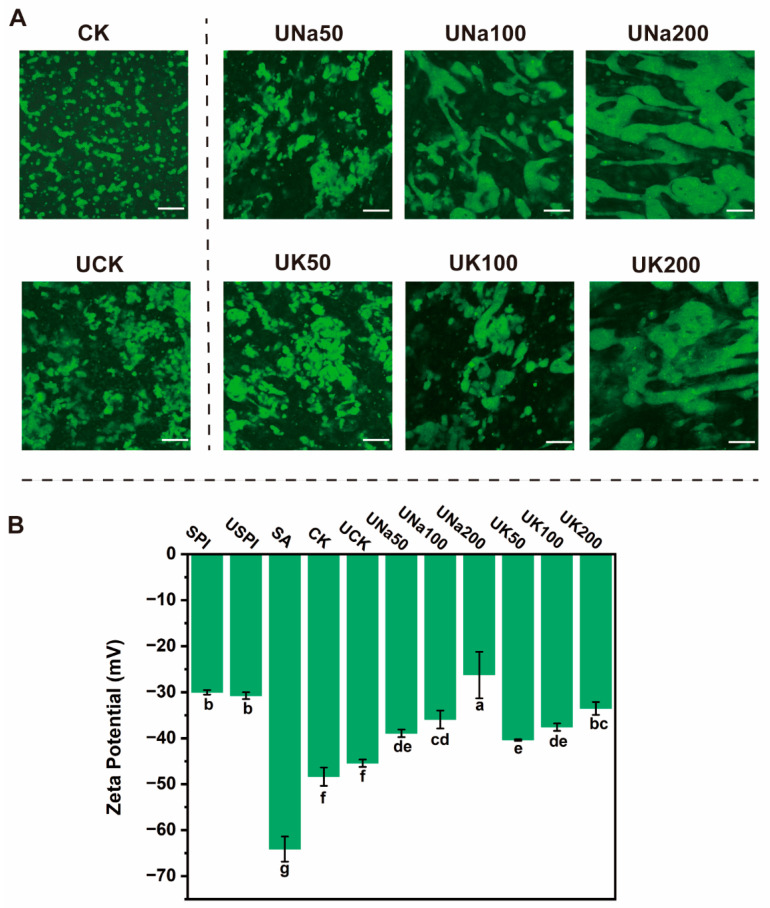
The confocal laser scanning microscopy images (**A**) and Zeta-potential (**B**) of SA/SPI composite solution (CK) and SA/USPI composite solutions (UCK) with different salt types (NaCl and KCl) and contents (50, 100, and 200 mM).

The microstructural analysis of composite solutions subjected to ultrasonic (US) treatment was conducted using confocal laser scanning microscopy (CLSM). Furthermore, the influence of salt ions on the morphological features of the solutions was investigated in Figure 2. The proteins were labeled with green fluorescent. As shown in Figure 2A, a discernible increase in protein aggregation occurred following US treatment when comparing CK and UCK, accompanied by no significant changes in surface charge (Figure 2B). The introduction of salt leads to reduction of absolute zeta potential value of the composite solution, attributable to the charge screening phenomenon, alongside the aggregation of proteins, driven by the ionic impact of salt on SPI’s surface charge [27]. Furthermore, the application of ultrasonic treatment intensified aggregation protein was further prompted by salt addition, the US treatment resulted in a greater exposure of proteins with negative charges on the surface [28,29], consequently elevating the protein’s negative surface charge. Therefore, the interaction between the modified SPI’s amino groups and SA’s carboxyl groups was amplified by the salt, leading to intense protein aggregation (UNa200, UK200).

#### 3.1.2. Rheological Properties Analysis

**Figure 3:**
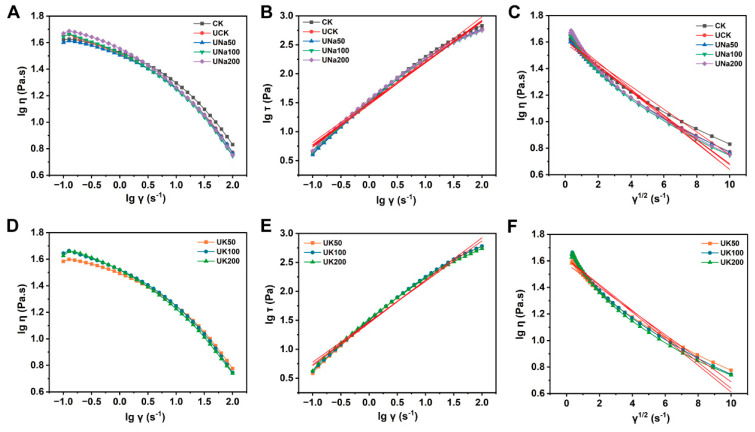
Plotted fits of lgη−lgγ (**A**), lgτ−lgγ (**B**), lgη−γ^1/2^ (**C**) of CK, UCK, and SA/USPI composite solutions with different NaCl contents; Plotted fits of lgη−lgγ (**D**), lgτ−lgγ (**E**), lgη−γ^1/2^ (**F**) of SA/USPI composite solutions with different KCl contents.

The rheological properties of SA/USPI composite solution were studied to investigate the effect of ultrasound pre-treatment and salt types/contents. Figure 3A,D demonstrate the effect of these factors on the viscosity of the solution at different shear rates. This indicates that the solution exhibits a typical shear-thinning behavior. The non-Newtonian index could be fit by Figure 3B,E and calculated using Equation (1). As indicated in Table 1, all samples exhibited a pseudoplastic fluid behavior (n < 1). Compared between sample CK and UCK, the ultrasound treatment resulted in an increase in the non-Newtonian index, indicating an enhancement in fluidity [30,31], which would be beneficial for the fiber spinning process. Additionally, increasing the salt content decreased the non-Newtonian index, implying that the presence of salt ions impacts the fluidity of the spinning solution, which continues to affect the spinning process later on [27]. 

In order to further characterize the spinnability of the spinning solution Figure 3C,F. The structural viscosity index (△η) was calculated through Equation (3), and it can be used to characterize the structurization of a spinning solution, where a small △η indicates a low structurization but better spinnability and fiber quality [30,31,32,33,34]. In Table 1, the △η of sample CK was much higher than sample UCK which implies that the ultrasound treatment causes a low structurization and better spinnability of the fiber. Furthermore, the incorporation of salt into the solution resulted in an observable increase in Δη. This phenomenon can be attributed primarily to electrostatic screening, which effectively mitigates the electrostatic repulsion forces acting between the constituent protein molecules [35,36,37], therefore, as salt content increases, the composite solution obtains lower spinnability which further affects the coagulation process.

### 3.2. Effect of Ultrasound Treatment and Salt on the SA/USPI Composite Fiber Characterization 

Through wet spinning, the composite solution is spun into a coagulation bath, culminating in the creation of a fiber structure. Varied formulations and proportions of the composite solution result in different characteristics of the fibers.

#### 3.2.1. The Micro-Morphology of SA/USPI Composite Fiber 

**Figure 4:**
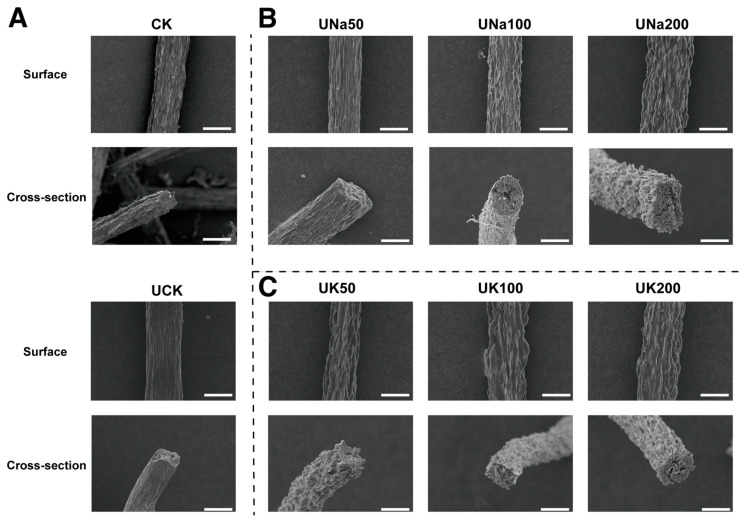
The SEM images (**A**) of the surface and cross-section of SA/SPI composite fibers with and without ultrasound treatment on SPI and these fibers with different NaCl (**B**) and KCl (**C**) contents. Scale bar: 50 μm.

The SEM images of SA/SPI composite fibers with and without ultrasound treatment, under different NaCl and KCl content, were presented in Figure 4. The surface morphology exhibits a distinctive grooved structure along the fiber with varying depths on all samples. This phenomenon was caused by the dual diffusion−reaction mechanism during the process of wet spinning [7,38]. The cross-sectional diameter of all obtained fibers consistently measured approximately 60 µm. Notably, within the coagulation bath, calcium ions interact with both SA and SPI components immediately upon the entry of the composite solution, resulting in ion exchange and the formation of crosslinks with SA, thereby establishing a surface gel layer [38]. The Ca^2+^ continues to diffuse to the center, whereas the reaction between SA and Ca^2+^ creates a junction, forces out the water molecules and leads to uneven fiber shrinkage that presents a porous structure (Figure 4A) [23,39]. Ultrasound treatment on SPI however, changes protein solubility by changing the conformation and structure of the protein which reduces the size of SPI protein molecules through cavitation forces and the hydrophilic parts of the amino acid would open up towards the water [17,28,40]. This engages the aggregation of protein during coagulation. The UCK fiber ends up with less groove, less porous structure (Figure 4A), and fewer filaments compared to sample CK, which might be attributed to the tension received during the fiber coagulation process; the weaker fiber structure would be more prone to breakage. 

With the addition of salt, the groovy texture on the fiber surface intensified and witnessed a more porous structure from the fiber cross-section (Figure 4B,C). Due to the process of dual diffusion reaction during fiber coagulation, salt ions in the spinning solution diffuse into the coagulation bath, the pressure at the center of the fiber decreases, and the pressure difference pushes the outer surface to the center of the fiber result in strip and groove appearance on the surface of the fiber [39]. As salt content increases, the salt ions drift out of the fiber faster due to the large concentration gradient, which results in a large pressure difference between the center and the surface of the fiber [23]. This intensifies the shrinkage and causes more groovy texture on the surface of the fiber. 

#### 3.2.2. The Secondary Structure of SA/USPI Composite Fibers

**Figure 5:**
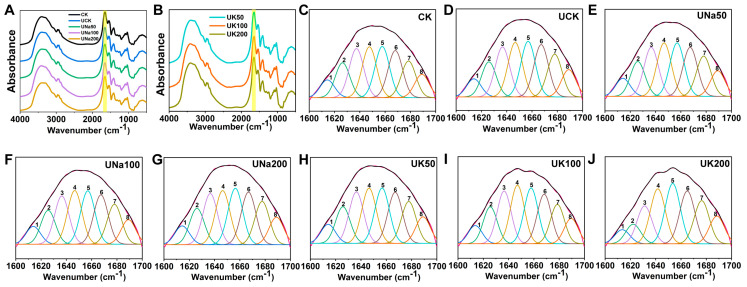
FT-IR spectrum (**A**,**B**) of SA/SPI (control) and the SA/USPI composite fibers with different salt types and contents; (**C**) refers to Gaussian peak fitting of SA/SPI composite fibers (control) (**D**) refers to Gaussian peak fitting of SA/USPI composite fibers; (**E**–**G**) refer to Gaussian peak fitting of SA/USPI composite fibers with 50 mM NaCl, 100 mM NaCl, and 200 mM NaCl; (**H**–**J**) refer to Gaussian peak fitting of SA/USPI composite fibers with 50 mM KCl, 100 mM KCl, and 200 mM KCl.

The structure change in protein has been investigated through FT-IR by analyzing the changes in peak position. The deconvolution of amide I (1600–1700 cm^−1^) provides essential details about secondary structures presented as α-helix, β-sheet, and random coil [22,41,42]. Within region amide I, it was divided into eight peaks as shown in Figure 5C to I. The absorption peak 1–3 (1610–1640 cm^−1^) represents the β-sheet, peak 4 (1640–1650 cm^−1^) represents the random coil, and peak 5 (1650–1660 cm^−1^) represents the α-helix [41,42,43,44]. The β-sheet content of the composite fiber decreased, and both the α-helix and random coil contents increased. The α-helix structure is related to protein folding, where a higher content of α-helix might adopt more interaction between the SPI and SA, and higher β-sheet content indicates better mechanical properties of the composite fiber [8,22,41]. The relative content of the secondary structure was delivered through peak calculation as shown in Table 2. Compared to related studies on SPI/SA composite fiber characterization [27], the US treatment on SPI alters the protein structure, resulting in higher β-sheet content but lower α-helix and random coil. In combination with the results in macroscopic phase behavior images, zeta potential, and the SEM images of the composite fiber’s surface and cross-sections, after ultrasound treatment, the composite solution exhibits an elevated level of aggregated protein molecules. This phenomenon can be attributed to the fact that the soy protein isolate (SPI) experiences a significant relaxation in its molecular structure subsequent to ultrasound treatment. As stated by Jambrak et al. [40], the presence of cavitation phenomena induces the disruption of hydrogen and hydrophobic bonds within the SPI, leading to a reduction in the molecular weight of the protein. This disruption fosters an enhancement in the interaction between proteins and water molecules, resulting in a greater fraction of the protein’s surface becoming enveloped by water molecules. therefore, higher protein solubility within the composite solution and an increase in the repulsive forces between the SA and SPI. Later within the coagulation bath, a higher number of calcium ions was able to interact with SA, resulting in a higher degree of structure aggregation. Consequently, in comparison to SA/SPI composite fibers that have not undergone ultrasound treatment, the ultrasonication process contributes to an elevation in the mechanical properties of the composite fibers.

In the presence of salt within the system, a persistent decline in β-sheet content is observed, concomitant with an increase in α-helix content as the salt concentration increases. This observation serves to elucidate the impact of salt ions on the intermolecular interactions between SA and USPI [27], consequently leading to diminished mechanical properties of the composite fiber.

#### 3.2.3. The Thermal Stability of SA/USPI Composite Fibers

**Figure 6:**
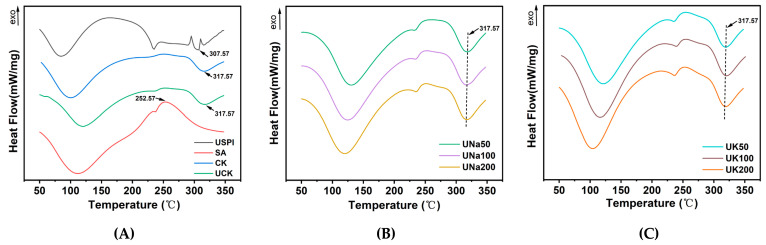
DSC (**A**) curves of SA, USPI, SA/SPI composite fibers (CK), SA/USPI composite fiber (UCK); DSC (**B**) curves of SA/USPI composite fibers with 50 mM NaCl, 100 mM NaCl, and 200 mM NaCl; DSC (**C**) curves of SA/USPI composite fibers with 50 mM KCl, 100 mM KCl, and 200 mM KCl.

The thermal stability of the sample is elucidated through DSC analysis. In order to satisfy the prerequisites for subjecting the product to elevated temperatures during cooking, an assessment of the thermal stability of the composite fiber was conducted. As illustrated in Figure 6A, sample USPI exhibited an endothermic peak at 87.00 °C, indicative of the initiation of water evaporation. The endothermic peak observed at 307.57 °C corresponds to the thermal decomposition of the USPI. And for the pure SA fiber exhibits an exothermal peak at 252.57 °C, signifying its thermal decomposition. Notably, the UCK fiber exhibits an endothermic peak at 317.57 ± 1 °C, mirroring the behavior of sample CK, thereby implying that ultrasound treatment does not exert a significant influence on the thermal stability of the fiber. Combining this finding with the observations presented in Figure 6B,C, it is evident that all samples manifest endothermic peaks at 317.57 ± 1 °C. Consequently, it can be concluded that neither the type nor the content of the added salt has a significant impact on the thermal stability of SA/USPI composite fibers.

#### 3.2.4. The Water-Holding Capacity of SA/USPI Composite Fibers

**Figure 7:**
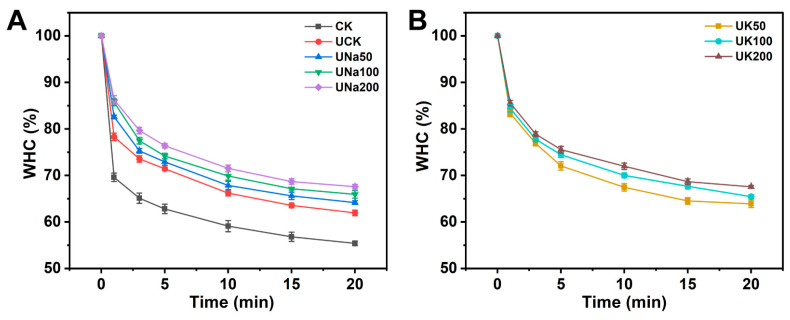
The WHC (**A**) of SA/SPI composite fiber (CK), SA/USPI composite fibers (UCK), and SA/USPI fibers with 50 mM, 100 mM, and 200 mM NaCl; the WHC (**B**) of SA/USPI composite fibers with 50 mM, 100 mM, and 200 mM KCl.

A successful meat analog product would need to fulfill the criterium of relatively high water retainability in order to constitute a juicy product [45,46,47]; the water-holding capacity of the SA/USPI composite fiber with different salt types and contents was analyzed and the results are shown in Figure 7. Comparing sample CK and sample UCK, the US treatment increased more than 5% WHC of the fiber, which corroborates the result in SEM that the porous structure allows the fiber to entrap more water within the fiber structure. When salt was introduced to the system, the SA/USPI composite fiber’s WHC increased, regardless of the salt type. Therefore, upon introducing salt into the system, the fiber obtains higher water retainability, which would effectively improve the juiciness of the composite fiber.

#### 3.2.5. The Moisture Distribution of SA/USPI Composite Fibers

**Figure 8:**
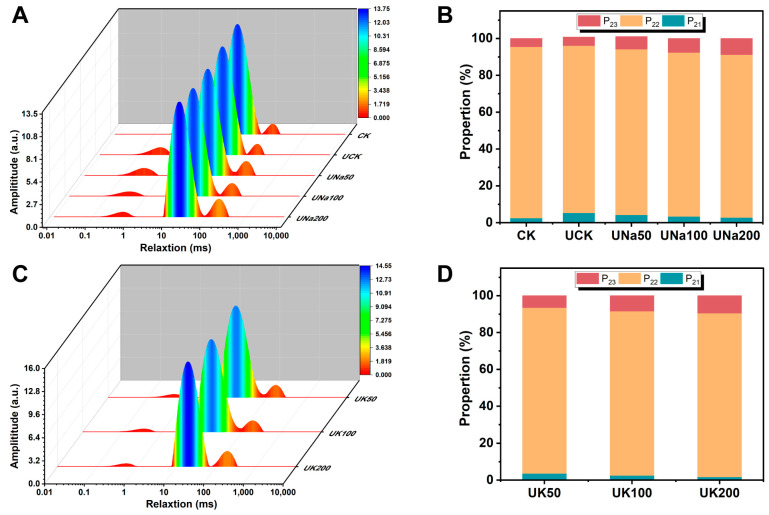
Distributed T2 relaxation times (**A**) and the proportion of water distribution (**B**) of SA/SPI composite fibers (CK), SA/USPI (UCK) and SA/USPI fibers with 0 mM, 50 mM, 100 mM, and 200 mM NaCl; distributed T2 relaxation times (**C**) and the proportion of water distribution (**D**) of SA/USPI composite fibers with 50 mM, 100 mM, and 200 mM KCl.

An analysis performed by LF-NMR aimed to investigate the distribution and proportion of different states of water in the fiber system [48]. The T_21_ (0.1–10 ms, bound water), T_22_ (10–100 ms, immobile water), and T_23_ (100–1000 ms, free water) in Figure 8A,C were the transverse relaxation times from short to long; the water component with less fluidity corresponds to a shorter relaxation time, whereas an increased relaxation time refers to a greater fluidity of water components [49,50]. The P_21_, P_22_, and P_23_ correspond to the proportion of water distribution. As shown in Figure 8B,D, after the US treatment, sample UCK showed a higher proportion of bound water than sample CK, whereas the proportion of free water presents no apparent difference. This further claims the result in the fiber’s water-holding capacity analysis that US treatment improves the fiber’s water retainability [51], where sample CK’s WHC had a large gap difference compared to other fibers with US treatment. 

When a different content of salt was added, the proportion of water states changed. Samples containing different salt types exhibited a consistent pattern, wherein P_23_ exhibited an increase as salt content increased, and P_21_ demonstrated a concurrent decrease with increasing salt concentration. Indicating alterations of the water mobility in the composite fiber, it was supposed that the free water (T_23_) increment was due to the formation of a porous structure, which would trap more water in the fiber structure. Therefore, with greater salt addition, the fiber retains an enhanced porous structure, thereby contributing to improved water retention, and would have the potential of increase the juiciness to the fiber. 

#### 3.2.6. The Mechanical Analysis of SA/USPI Composite Fibers

**Figure 9:**
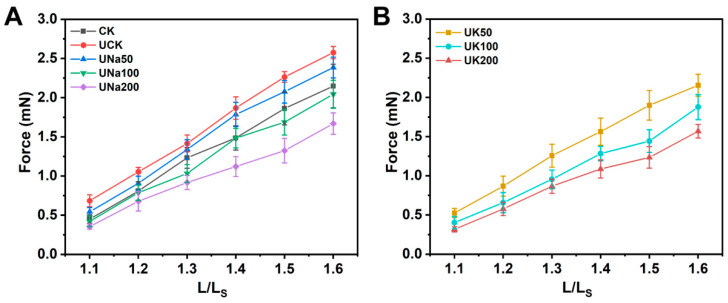
The tensile strength (**A**) of SA/SPI composite fibers (control) and SA/USPI composite fibers with 0 mM, 50 mM, 100 mM, and 200 mM NaCl; the tensile strength (**B**) of SA/USPI composite fibers with 50 mM, 100 mM, and 200 mM KCl.

The mechanical properties of the composite fiber were conducted, with results shown in Figure 9. The tensile force of the fiber decreased with the increase in salt content. This would correspond to the salt ions eliminating the reaction between Ca^2+^ with SA during the coagulation process [27]. Combined with the result images from SEM analysis, with only the US treatment, the UCK fiber will show a more aggregated protein structure than other fibers with salt added. After salt addition, as the salt content increases, the tensile strength of the fiber reduced, further confirming the result in FT-IR, and the structure of these fibers appeared more porous than the CK fiber and UCK fiber, which is consistent with the result shown in the SEM image. The type of salt used shows no viable difference in the mechanical properties of the composite fiber.

## 4. Conclusions

In this study, the effect of ultrasound treatment and salt on the SA/USPI composite system, and meat analog fiber quality after being processed through wet spinning was investigated. The ultrasound treatment promotes protein aggregation, and with the addition of salt, the protein aggregates even more as salt content increases, which results in a low structural viscosity index and better spinnability. The Ca^2+^ interacts with SA during the coagulation process, and the engagement of ultrasound causes the hydrophilic parts of the amino acid in protein to open up towards the water, which ends up with a more porous structure. The porous structure and the hollow in the center of the fiber significantly increase the water-holding capacity of the fiber. With only the effect of ultrasound, the fiber obtains higher water-holding capacity and better mechanical properties, while remaining a similar thermal stability. Regardless of the type of salt added to the system, with higher salt content, the thermal stability remains stable. As salt content increases, the water-holding capacity will be enhanced and the mechanical properties decline. Hence, this work would not only enrich the details of preparing plant-based meat analog fibers through wet-spinning but also provide possibilities for the production of different meat parts.

## Figures and Tables

**Table 1:** The non-Newtonian (n) and structural viscosity index (△η) of control and SA/USPI composite solutions with different salt types and contents.

Sample	n	$R\begin{array}{c} 2\\ 1 \end{array}$	△η	$R\begin{array}{c} 2\\ 2 \end{array}$
CK	0.7464	0.99	9.345	0.98
UCK	0.7797	0.99	8.602	0.98
UNa50	0.7311	0.99	9.099	0.98
UNa100	0.7073	0.99	9.777	0.97
UNa200	0.6971	0.99	10.098	0.96
UK50	0.7371	0.99	8.889	0.98
UK100	0.7297	0.99	9.777	0.97
UK200	0.6978	0.99	10.011	0.96

All tests were conducted at 25 °C. Parameters (n, △η) are obtained using Equations (1)–(3), respectively. R$\begin{array}{c} 2\\ 1 \end{array}$ and R$\begin{array}{c} 2\\ 2 \end{array}$ represent the degree of fitting of the curves.

**Table 2:** Fitting results of the secondary structure of control and SA/USPI composite fibers with different salt types and contents.

Sample	α-Helix	β-Sheet	Random Coil
CK	16.65	29.30	17.25
UCK	16.03	31.27	15.95
UNa50	16.54	31.55	16.54
UNa100	16.87	30.19	17.07
UNa200	17.06	28.80	17.53
UK50	16.44	32.09	16.40
UK100	16.91	30.79	17.45
UK200	17.49	28.05	17.98

## Data Availability

Data are contained within the article.
